# Concordance between Ov16 Rapid Diagnostic Test(RDT) and Ov16 Enzyme-Linked Immunosorbent Assay (ELISA) for the Diagnosis of Onchocerciasis in Areas of Contrasting Endemicity in Cameroon

**DOI:** 10.1016/j.parepi.2023.e00290

**Published:** 2023-05

**Authors:** Relindis Ekanya, Amuam Andrew Beng, Muwah Anastacia Anim, Yokyu Zachary Pangwoh, Obie Elisabeth Dibando, Narcisse Victor Tchamatchoua Gandjui, Abong Raphael Awah, Glory N Amambo, Gordon Takop Nchanji, Bertrand Lontum Ndzeshang, Theobald Mue Nji, Fanny Fri Fombad, Abdel Jelil Njouendou, Esum Mathias Eyong, Jerome Fru Cho, Peter A Eyong, Kebede Deribe, Ntonifor Helen Ngum, Allison Golden, Samuel Wanji

**Affiliations:** 1 Parasite and vector biology research unit (PAVBRU), Department of Microbiology and Parasitology, Faculty of Science, University of Buea, Buea, Cameroon; 2 Research Foundation in Tropical Diseases and Environment, (REFOTDE), Buea, Cameroon; 3 Department of Biological Sciences, University of Bamenda, Cameroon; 4 PATH, Seatle, USA; 5 Children’s Investment Fund Foundation, Addis Ababa, Ethiopia; 6 Brighton and Sussex Medical School, Department of Global Health and Infection, Brighton, UK

**Keywords:** Onchocerciasis, agreement, antibodies test, contrasting endemicity

## Abstract

The diagnosis of onchocerciasis in endemic areas has been demanding given the need to replace the invasive skin snip method with a more sensitive and specific rapid point-of-contact tool. Filarial antigen detection tests are better alternative methods in diagnosing *Onchocercal* infections, as they detect infections and could be used to monitor transmission in endemic areas following mass drug administration. With the shift in paradigme from control to elimination, a rapid point- of-contact tool is required to support elimination programs. This was a cross-sectional, community-based study conducted in 50 villages selected from six health districts using a systematic sampling technique. Individuals ≥17 years who had lived in the community for a duration of 5 years and above provided blood specimens for IgG4 antibodies testing against *O. volvulus* antigens. Data were analyzed using SPSS v.20 and expectation maximization to classify optical densities for positive and negative samples from ELISA results. The kappa statistics was used to measure the level of agreement between the two tests. In a total of 5001 participants which were recruited for the study, 4416 (88.3 %) participant samples passed the plate quality control criteria and were considered for the test comparison analysis. Out of the 4416 participants, 292 (6.6 %) tested positive with Ov16 RDT and 310 (7.0 %) with Ov16 ELISA. All those who tested positive with the rapid test agreed positive with ELISA. The overall percentage agreement was 99.2 %, the Kappa score of 0.936. The results obtained indicate an excellent agreement between ELISA and RDT as measured by kappa (0.936) which was statistically significant (**P<0.001**). Our experience with the Ov16 ELISA biplex rapid test was favorable. However, the Ov16 RDT test may be more appropriate to use in remote areas for the point diagnosis of onchocerciasis in view towards achieving elimination in Africa.

## 1 Introduction

Onchocerciasis is a disease caused by a tissue dwelling parasitic nematode worm *Onchocerca volvulus*. The disease is endemic mainly in tropical Africa extending to Central, South America and the Arabian Peninsula, particularly in Yemen. An estimated 18 million people are infected with *O. volvulus* worldwide with 99 % in 31 West and Central African countries [1]

The World Health Organisation in collaboration with the African Program for Onchocerciasis Control (APOC) recommended repeated rounds of mass drug administration (MDA) with ivermectin (anti-filarial) donated by Merck and co Inc, to interrupt transmission of the nematode worm that causes onchocerciasis [2]. While the drug may kill the microfilaria and reduce transmission, it does not completely kill the adult worms. Therefore community drug administration must continue for years in order to eliminate the parasite and risk of infection.

Despite this, in Cameroon onchocerciasis is still endemic in almost all regions after several years of ivermectin MDA, and a probable non-interruption of transmission of the disease[3,4].A variety of techniques have been used to assess the impact of control measures. [5]. Diagnosis relies on the use of skin biopsy to detect microfilaria in the subcutaneous area of the upper iliac crest of a study population. This method is invasive and not very sensitive especially when microfilarial (mf) skin densities are low[6].

Recent studies suggest that, serological tests (antibody-based assays) avoid some of the problems associated with microfilaria and DNA detection because of their ability to identify both past and current infections without dependency on the timing of MDA[7]. Commercial kits utilizing specific monoclonal antibodies to detect *O. volvulus* antigens are currently available. An Enzyme-Linked Immunosorbent Assay (ELISA plate test), has been used to diagnose microfilaremic and amicrofilaremic individuals with high sensitivity and specificity in foci that received extensive rounds of MDA[8]. However, the requirement for a laboratory capable of performing ELISA and the variations in test protocol has limited the utility of this test [9,10]Recently a new Ov16 rapid diagnostic test (RDT) SD BIOLINE Oncho/Lf IgG4 biplex rapid test was developed using IgG4 monoclonal antibodies with high sensitivity and specificity[11]. The Ov16 RDT is a promising field friendly tool due to its ease to use and could be applied to a large population for mapping and even post-MDA surveillance[7,11].

The development of a highly sensitive and specific point-of-care tool to detect the presence of human IgG4 antibodies specific to the recombinant antigen Ov16 is an important goal towards improving the diagnosis of human onchocerciasis. Given the need to replace the painful skin snipping method, the antibody assay was developed to enhance continuous monitoring and evaluation to achieve the global goal towards the elimination of onchocerciasis by 2025. The Ov-16 serology with IgG4 has been a major advancement in the fight towards the elimination of onchocerciasis[12]. It has been used to confirm elimination of the onchocerciasis in the Americas and recently in Africa[13,14]The Ov-16 antigen demonstrated good performance for the diagnosis of onchocerciasis although some crossreactions with *Mansonella ozzardi* which is not present in Africa was reported[12]. Recent studies demonstrated the feasibility of implementing the Ov16 rapid test into ongoing onchocerciasis control programs[15].

Both Ov16 tests (a lateral flow rapid diagnostic test (Ov16 RDT) and a laboratory based test (Ov16 ELISA) have been used recently to diagnose onchocerciasis in areas that receive MDA[8,15]. There exists little knowledge on the level of concordance between the two Ov16-based tests. This study aims at comparing a lateral flow rapid diagnostic test (Ov16 RDT) to a laboratory based test (Ov16 ELISA) for the diagnosis of onchocerciasis in areas of contrasting endemicity in Cameroon.

## 2 Methods

### 2.1 Ethical Considerations

Ethical approval was obtained from the National Ethics Committee for Human Health Research (N° 2015/09/640/CE/CNER/SH/SP). A copy of the research protocol was submitted to the Ministry of Public Health for administrative clearance. In the study communities, details on the potential risks and benefits of the study were made known to the community leaders (Chiefs or Quarter-heads) and study participants using the participant information sheet. The recruitment of individuals was voluntary. Participants, who gave their consent voluntarily, signed the consent form. Participant who were not literate made fingerprint. If less than 21 years of age the verbal assent and permission were obtained from the study participant and parent/legal guardians. All participants’ information collected during the study were kept on a password-protected database and was strictly confidential. The participant’s identity and results were kept private and individual barcodes were used to label specimen and forms.

### 2.2 Study areas

**Figure 1:**
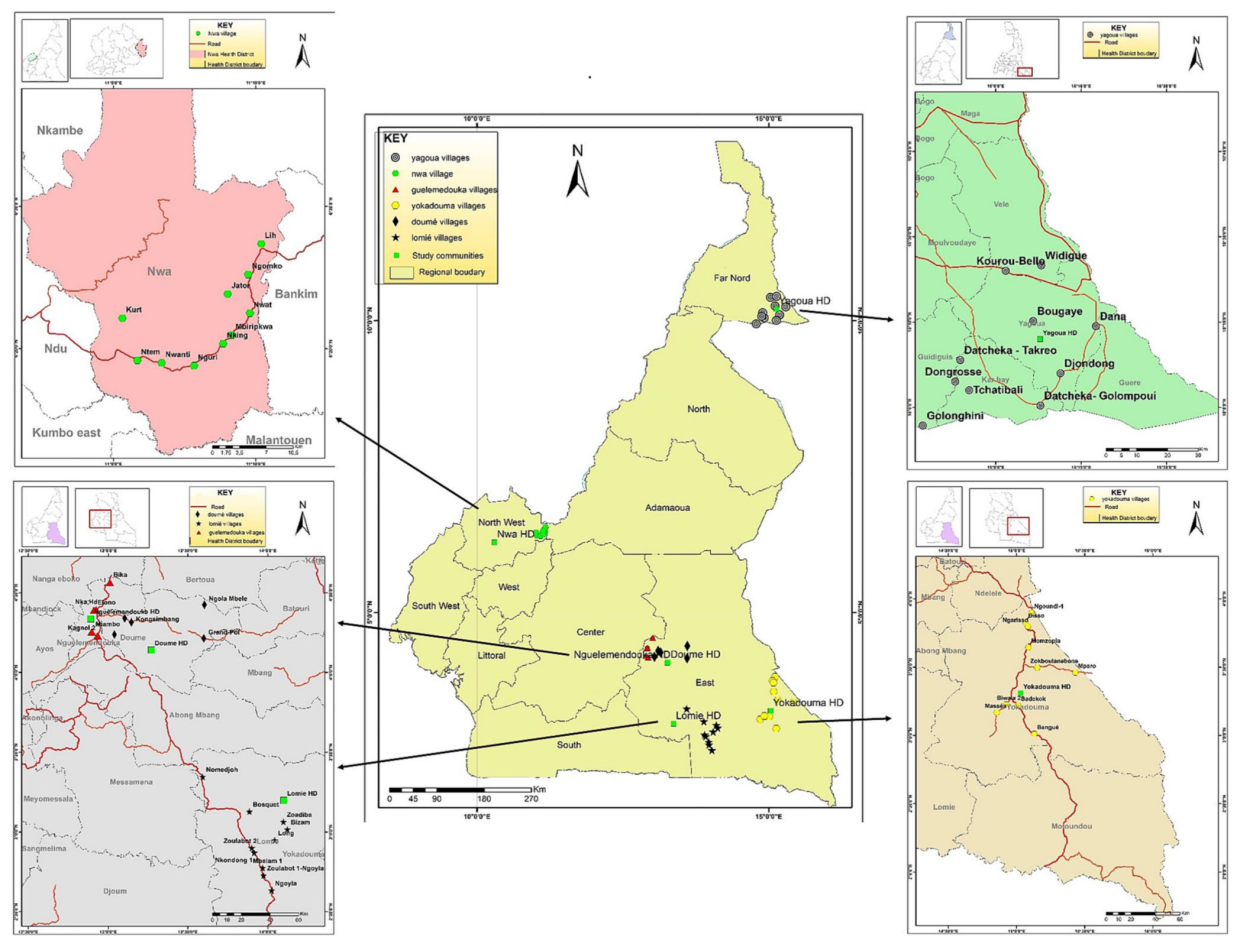
Map of surveyed communities from six health districts in Cameroon.

Overall, 50 villages were selected from six health districts in three regions of Cameroon (figure 1). Four health Districts from the East region (Doume, Lomie, Nguelemendouka and Yokadouma), Nwa health district from the North West region and the Yagoua Health Districts from the Far North region. The health districts have different levels of endemicity of *Onchocerca volvulus* with other filarial parasites such as *Loa loa,, and Mansonella perstans*.

### 2.3 Study design

**Figure 2:**
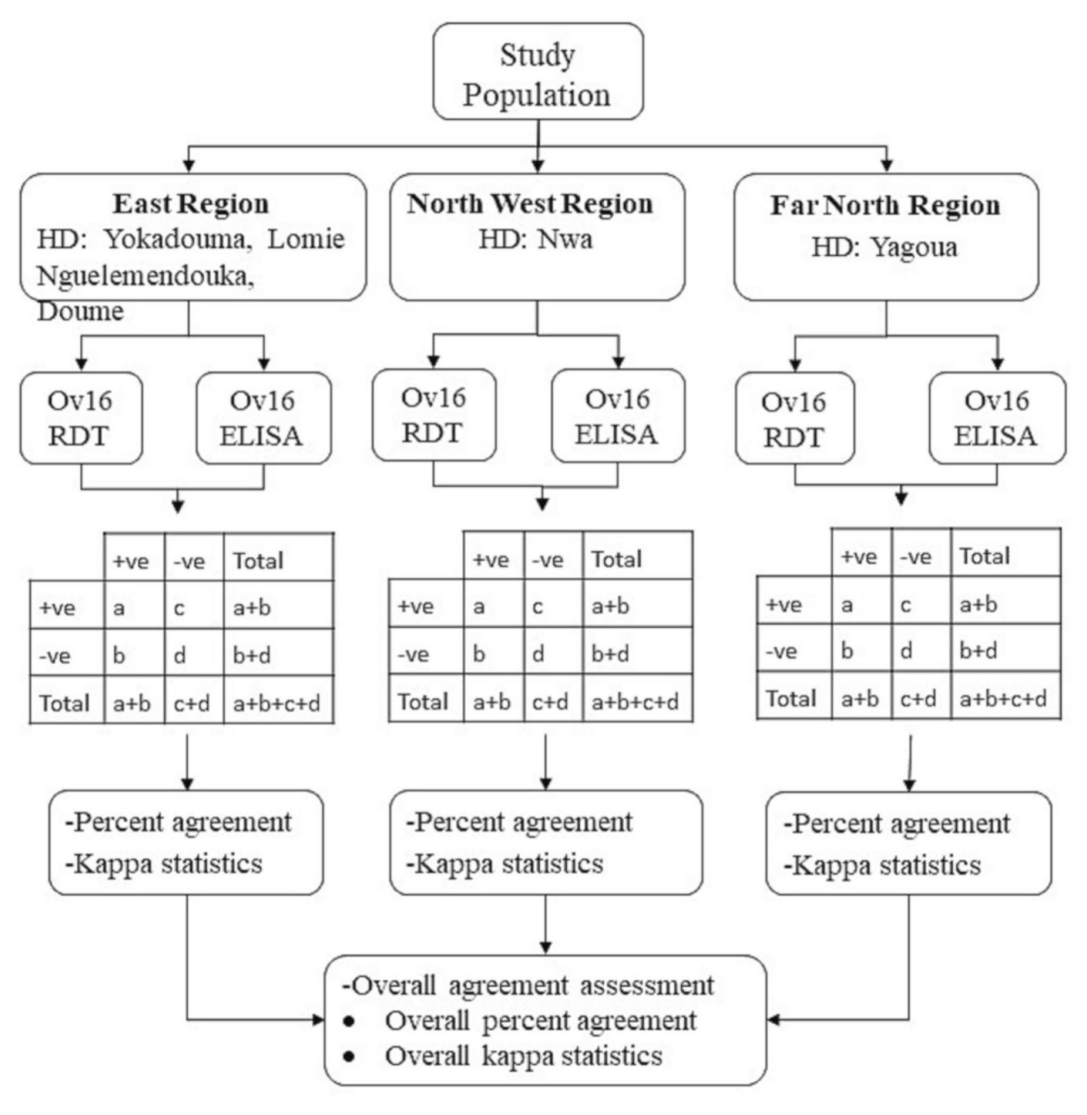
Schematic presentation of study design

The study was a cross-sectional and community based study design. Recruited volunteers that met the eligibility criteria gave their consent by signing the consent form. The parent or legal guardian provided consent for participants below 21 years. Demographic data from participants were collected and recorded on a structured questionnaire and this was followed by serological examination using the SD BIOLINE Oncho/Lf Biplex test kits for point diagnosis of onchocerciasis. In addition, dried blood spots were collected on TropBio filter papers for ELISA(figure 2).

### 2.4 Study Population/Sampling

The study participants were individuals of both sexes aged ≥ 17 years and who had lived in the community for at least five years. Based on the stratification of health districts, communities were selected from six health districts of Cameroon. Ten villages were randomly selected from each of the health districts except Doume and Nguelemendouka where 5 villages were selected in each health district because these health districts are small. In each village, a complete census of households was conducted and 50 households were selected using simple random sampling. In each household two or more volunteers ≥17 years who had lived in the community for at least 5 years were recruited.

### 2.5 Eligibility criteria

#### 2.5.1 Inclusion Criteria

Males and females aged ≥17 years and who had lived for at least 5 years in the study area, Individuals who gave their consent by signing the consent form.

#### 2.5.2 Exclusion Criteria

Males and females ≤17 years,individuals who had not lived in the community for up to 5 yearsand Individuals who did not sign the consent form.

### 2.6 Field Procedure using Ov16 Rapid Diagnostic Test (RDT)

The Rapid Diagnostic Test Alere™ SD BIOLINE oncho/LF IgG4 biplex test kit was used to detect the IgG4 antibodies specific to *Onchocerca volvolus* recombinant antigen (Ov16). The SD BIOLINE Oncho/Lf IgG4 biplex rapid test prototype manufactured by PATH (Program for Appropriate Technology in Health) and Standard Diagnostics (Yongin-si, Gyeonggi-do, South Korea).Each test kit contained 25 test devices with desiccant in individual foil pouch, assay diluents (1x3ml/vial), disposable capillary pipettes (10 uL), lancets, and alcohol swabs Using a lancet, a finger prick was given to consented individuals and the resulting blood collected using a 10 μL disposable micro-capillary pipette unto the sample well on the test device. Four drops of assay diluent (3ml/vial) were added to the assay-diluent well. Results were read and recorded after 30 minutes and the results from the strips remained stable for 24 hours.

### 2.7 Dried Blood Spots (DBS) Collection for ELISA

The remaining finger pricked blood above was collected using a 75 uL capillary tube and blotted on six protrusions of filter paper sections (CeLLabs’ TropBio filter papers). The filter papers were carefully placed on a drying rack and allowed to air dry at room temperature (RT). The dried CeLLabs’ TropBio papers were placed in separate envelops. The envelops (twenty-five cards per pouch) were placed in plastic bags and stored in zip-lock silver pouches containing one-unit clay desiccant packets (Desiccare, Reno, NV, USA) and a humidity indicator card. Dried blood spots (DBS) were brought to the Research Foundation in Tropical Disease and Environment Laboratory (REFOTDE) and stored at -20 °C until used for ELISA.

### 2.8 Horseradish Peroxidase (HRP)-developed Ov16 ELISA procedure

Parasite-specific IgG4 antibody to Ov16 antigen were done using a short form indirect ELISA procedure (PATH protocol for Ov16 ELISA of Dried Blood Spot TropBio, short form protocol 2017) with some modifications. Samples were cut from saturated circles (6 mm) of blood from TropBio (Whatman) paper and added to the elusion plate (Costar Assay plate, 96-well clear round bottom plate with lid) using clean tweezers. Pre-cut positive and negative controls were added to assigned wells followed by elution with 150 μL of 1 × (Phosphate-Buffered Saline-Tween (PBST) + 2% non-fat dry milk) to each well and incubated overnight at 4°C. Antigen coating was done by adding 100 μL of Ov16 antigen at final concentration (5 μL/mL in 1×PBS) per well to Immulon 2HB plate, covered with plate sealer and refrigerated overnight at 4°C[11].

The sample elutions were ran in duplicates in 96-well high binding polystyrene plates. The plates were washed 3 × with 1 × PBS-Tween, pH 7.4 (PBST, washing buffer) and blocked with 200 μL assay diluents and blocking buffer (PBST+ 5 % Fetal Bovine Serum (FBS) followed by 30 minutes at 37 °C incubation. After the 30 minutes incubation, plates were washed 3 times with wash buffer. 50 μL of the diluted DBS samples were added to the Immulon 2HB flat bottom microtitre plates in duplicate to the corresponding wells. Controls were added to their corresponding wells and incubated at 37 °C for one hour. After incubation, plates were quickly inverted over the sink to shake out liquid by tapping upside down on a paper towel to remove remaining liquid. Washing was done 3 times and bound antibodies (primary antibody) were detected by exposure to 50 μL Horseradish Peroxidase conjugated mouse anti-human IgG4 (HP6025) ratio 1:5,000 per well, followed by 37°C incubation for 1 hour at and wash as described above. Secondary antibody detection 1μl of goat anti-mouse HRP (GαM-HRP) were prepared in 10ml 1× PBST+5%FBS (ratio 1:10,000) and 50 μL were added per well and incubated at 37 °C for 1 hour. Substrate 3, 3’, 5, 5’,-Tetramethylbenzidine (TMB) of 100 μL were added to each well and allowed to develop at 37 °C RT in the dark for 6minutes. A blue colour reaction was observed due to the developing TMB solution. The enzymatic reaction was stopped by adding 50 μL of 1N HCL and inserted in the spectrophotometer or Human Reader (HS) and the values read at absorbance 450 nm.

#### 2.8.1 Interpretation of Ov16 ELISA Results

ELISA result classified positive if normalized absorbance greater than threshold found by expectation maximization, an interative technique to find the best fit of two variable Gaussian distributions to normalized ELISA data. The ELISA dataset of normalized ELISA values (sample average/plate low positive control average) comprised all samples that passed the quality criteria. Plate passed if controls met product insert criteria the low-positive plate control duplicate well difference was <0.125 absorbance units. Sample passed if both duplicate absorbance values were <0.2, or duplicate well absorbance percent difference <37% (difference value/duplicate well average). ELISA was repeated if initial results failed criteria. Repeated ELISA results were used to replace the initial results if passed criteria; otherwise samples were excluded from analysis[16].

### 2.9 Data Management and Analysis

Data from the field was collected using structured questionnaire. The data was checked for completeness and accuracy daily. The demographic and RDTs data were entered into an electronic template created in Epi Info (Center for Disease Control and Prevention, Atlanta, GA), then exported to Microsoft Cloud while ELISA data was entered on a Microsoft Excel template. After merging, the data was then cleaned and stored in password protected computer accessible only to the investigator.

Data analysis was performed using the Statistical Package for Social Sciences (SPSS v.20.0) (Armonk, NY: IBM Corp) and association in prevalence with RDT and ELISA were analyzed using Pearson Chi square. ArcGIS 9.3 software was employed to generate map of the study areas. The Gaussian mixture model, expectation maximization were used to classify Optical Densities (OD) for positive and negative samples from ELISA results. ELISA results are classified positive if normalized absorbance greater than threshold found by expectation maximization, an iterative technique to find the best fit of two variable Gaussian distributions to normalized ELISA data[16]. The Kappa statistics was used to determine the level of agreement between the tests. Chi-square test was used to determine the significance between the two tests at 95% confidence interval and P-value ≤0.05 were considered statistically significant.

## 3 Results

### 3.1 Socio-demographic characteristics of study participants according to study areas

A total of 5001 participants were recruited for the study. Of this total, 4416 (88.3 %) participant samples with valid result passed the plate quality control criteria (sample average/plate low positive control average) were considered for the test comparison analysis.

**Figure 3:**
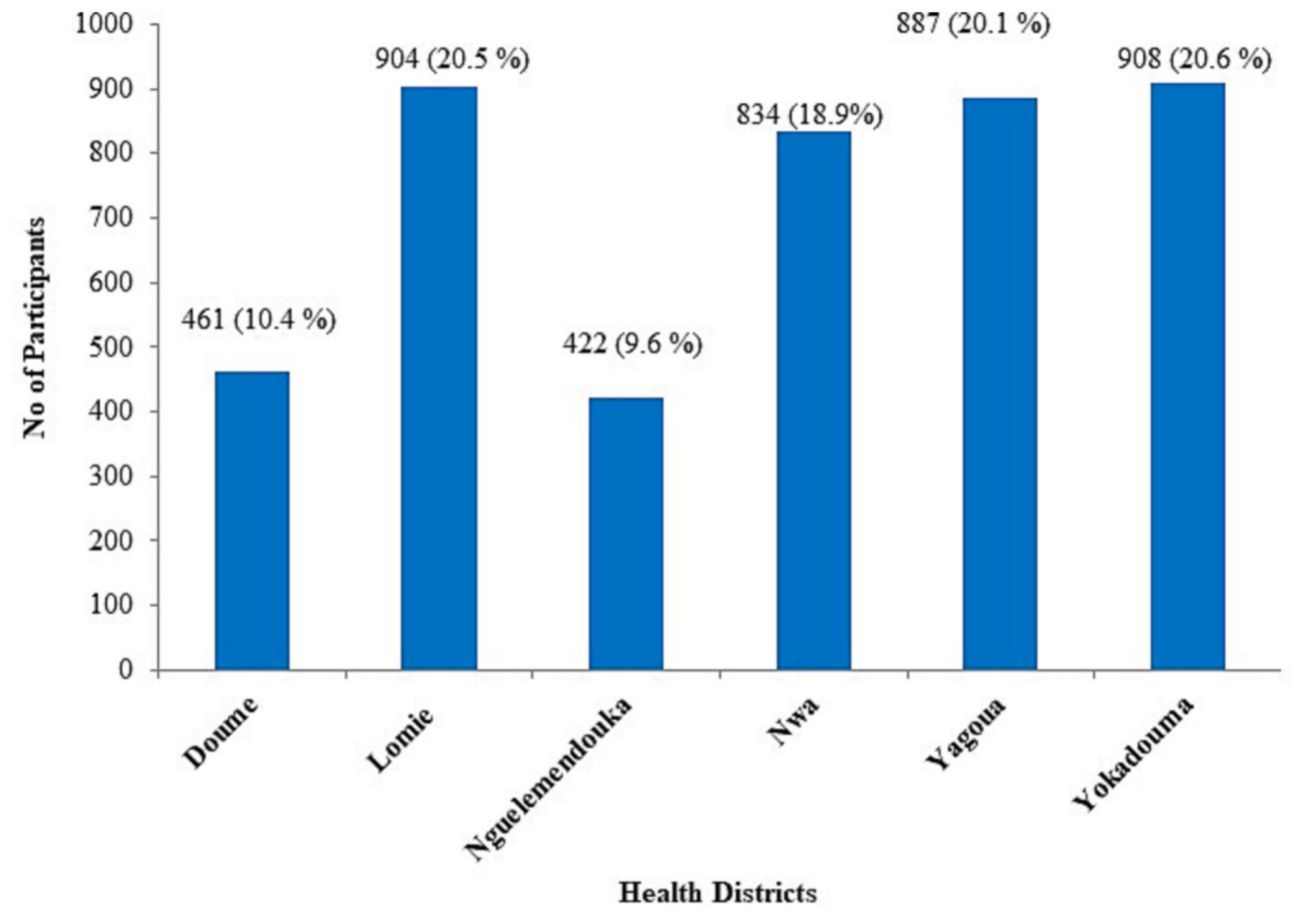
Distribution of study participants according to health districts

From the six health districts, Doume and Nguelemendouka had the lowest number of participants compared to the other health districts because only 5 villages were selected from each of these health districts while the others have a similar trend in the number of participants recruited (figure 3).

#### 3.1.1 Sex, age and duration in the community distribution of the study participants

Of the 4416 participants, 2036 (46.1 %) were males and 2380 (53.9 %) were females. Majority 1896 (42.9 %) of them had ages between 17-30 years while a minority 518 (11.7%) had ages between 51-60 years with a mean age of 38.70±18.326. A greater majority of the study participants 1218 (27.6 %) had stayed in the community between 16-25 years while a minority 289 (6.5 %) had stayed between 56-65years giving a mean duration of 30.77±20.236 (Table 1).

### 3.2 Previous History of Ivermectin Mass Drug Administration by Health Districts

A vast majority of participants from the East (Doume 461/461(100%), Ngelemendouka 324/422(76.7%) and Yokadouma 827/908(91.92%) Health Districts) declared they had never taken ivermectin except Lomie (East) 619/904(68.53%) where a majority of the participants said they have taken ivermectin. Also, all participants in the Doume461/461(100%) Health District declared they had never taken ivermectin. The Health Districts of Nwa and Yagoua recorded the highest number of participants 752/834(90.17%) and 790/887(89%) respectively who had taken ivermectin.

### 3.3 Prevalence of Onchocerciasis using Ov16 RDT and Ov16 ELISA in different health district

From the total 4416 participant that were considered for the test comparison analysis using the Alere™ SD BIOLINE oncho/LF IgG4 biplex rapid test and DBS for ELISA. The overall sero-prevalence was 6.6 % with the rapid diagnostic test and 7.0 % with the laboratory ELISA. The Nwa Health District had the highest sero-prevalence 25.5 % and 27.5% with the rapid diagnostic test and ELISA respectively (Table 2). The difference in the prevalence between the Ov16-based diagnostic tests and the health districts were statistically significant (**P = <0.001**)

### 3.4 Sero-prevalence of onchocerciasis using the Ov16 rapid diagnostic test and Ov16 ELISA according to socio-demographic characteristics of study participants

There were more positive females 152 (6.9 %) than males 140 (6.4 %). The sero-prevalence was higher in males (140/2036) 6.9 % with RDT and (152/2036) 7.5 % with ELISA compared to females (Table 3). The difference in the prevalence between the rapid test and ELISA among gender was not statistically significant.

The age class 31-40 recorded had the highest sero-prevalence 9.1 % and 9.4 % with Ov16 RDT and Ov16 ELISA respectively and the age class 17–30 had the lowest 5.1 % and 5.4 % with Ov16 RDT and Ov16 ELISA respectively. The difference in the prevalence between the rapid test and ELISA among age groups were statistically significant (**P = 0.004**).

Participants who had lived in the community between 36-45 years had the highest sero-prevalence 8.1 % with the rapid biplex test meanwhile with ELISA, the highest sero-prevalence 8.3 % was between 56-65 years duration in the community. The difference between duration in community and prevalence was statistically not significant for both Ov16-based tests.

The prevalence was low 2.1 % and 2.4 % with RDT and ELISA respectively among individuals who have never taken ivermectin compared to those who had taken ivermectin 10.6 % and 11.2 % with the rapid test and ELISA respectively. The difference between history on mass drug administration and prevalence with both tests were statistically significant (**P<0.001**).

### 3.5 Level of agreement between Ov16 RDT and Ov16 ELISA in the diagnosis of onchocerciasis

#### 3.5.1 Level of agreement according to health districts

Out of the 461 participants from the Doume Health District, 5(1.08%) were positive with Ov16 ELISA and 5(1.08%) positive with Ov16 RDT. Also, all participants (456)(98.92%), who were negative with Ov16 ELISA, were also negative with the Ov16 RDT. The percent agreement for both test in this health district was 100.0 %, the kappa score was 1.000 indicating an excellent agreement between the two test which was statistically significant (**P<0.001**).

With regards to the Lomie Health District, 904 participants were examined. Out of this total, 72(7.96%) were positive with Ov16 ELISA, and 70(7.74%) with Ov16 RDT. 832(92.03%) participants tested negative with Ov16 ELISA and 831(91.92%) with Ov16 RDT. The percent agreement was 99.6 % and kappa score 0.969 indicating an excellent agreement which was statistically significant (**P<0.001**).

In the Nguelemendouka Health District, 422 were examined, 3(0.71%) were positive with Ov16 ELISA, 3(0.71%) positive with Ov16 RDT. 419(99.2%) negative with Ov16 ELISA and 419(99.2%) negative with Ov16 RDT. The percent agreement was 100.0 % kappa score was 1.000 indicating an excellent agreement which was statistically significant (**P = 0.006;** Table 4)

Furthermore, in the Nwa Health District, out of the 834 participants with valid samples subjected for comparison. 229(27.45%) were positive with Ov16 ELISA and 213(25.54%) with Ov16 RDT. Also, 605(72.54%) were negative with Ov16 ELISA and 597(71.58%) with Ov16 RDT. The percent agreement was 96.2 % kappa of 0.902 indicating a perfect agreement which was statistically significant (**P<0.001**).

In the Yagoua Health District, out of the 887 participants examined, 1(0.11%) was positive with Ov16 ELISA and 1(0.11%) with Ov16 RDTThe percent agreement was 100.0 %, kappa of 1.00 indicating an excellent agreement which was statistically significant (**P<0.001**).

In a similar manner, in the Yokadouma Health District, 908 were examined, none were positive with both Ov16 ELISA and Ov16 RDT. 908 were negative with both Ov16 ELISA and Ov16 RDT. The percent agreement was 100.0 % kappa of 0.000 indicating agreement due to chance which was statistically not significant.

#### 3.5.2 Overall percentage agreement between Ov16 RDT and Ov16 ELISA

Out of the 4416 participants that were considered for the test comparison analysis using the antibody tests Ov16 RDT and Ov16 ELISA, 310(7.01%) were positive with Ov16 ELISA and 292(6.61%) were positives with the Ov16 RDT. In a similar manner, 4106(92.98%) participant had negative results with Ov16 ELISA and 4097(92.77%) negative with the Ov16 rapid test. The overall test agreement was 283(6.405%) positives and 4096(92.75%) in negatives. The overall % agreement was 99.2 % (table 5). The overall Kappa score was 0.936 indicating an excellent perfect agreement which was statistically significant (**P<0.001**). In addition, the sensitivity and specificity were 91.3 % and 99.8 % respectively. The positive and negative predictive value was 96.9 % and 99.3 % respectively.

#### 3.5.3 Positive and negative percent agreement in test results of Ov16 RDT and Ov16 ELISA according to health district

**Figure 4:**
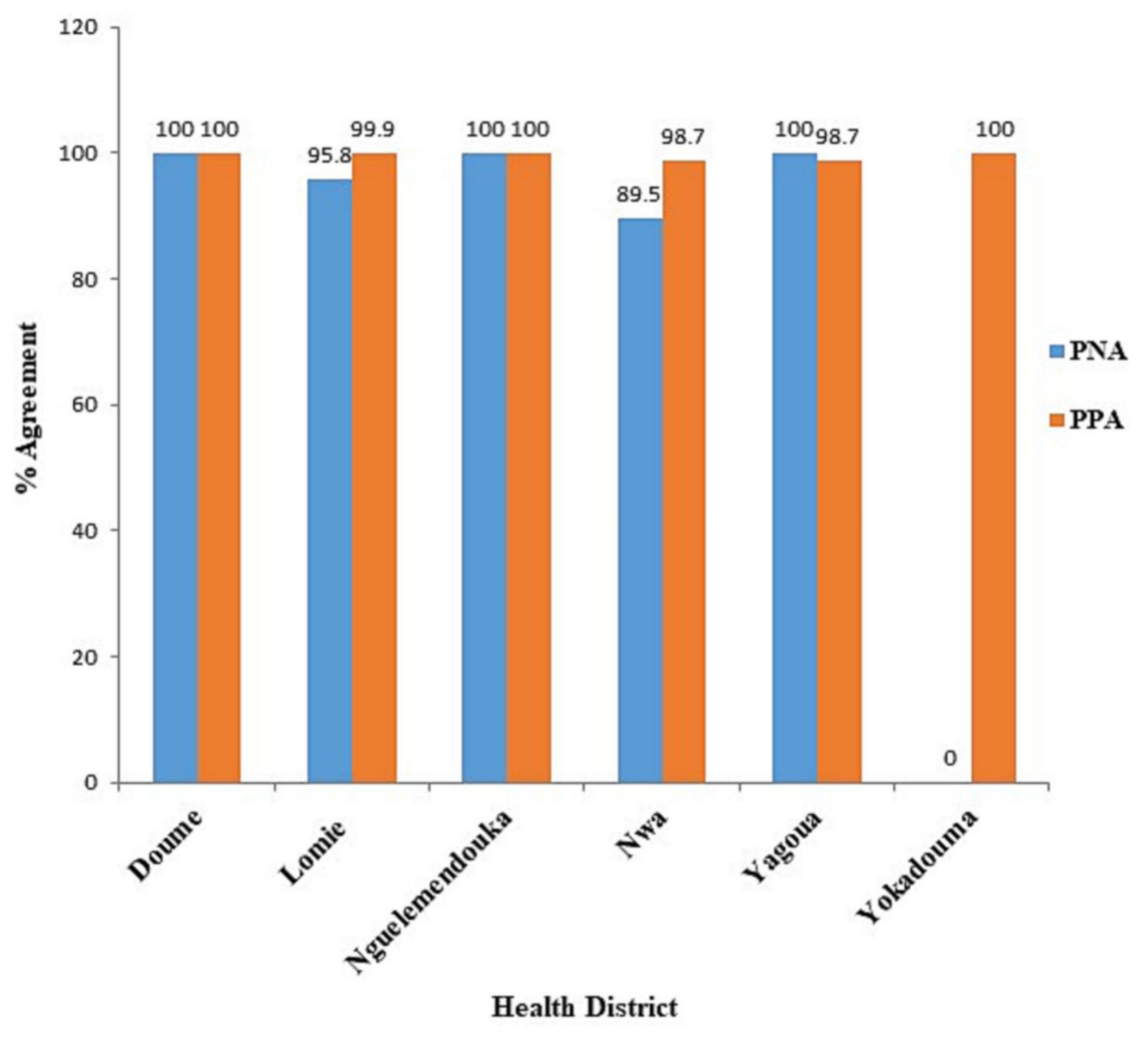
Percent positive and negative agreements in test results of Ov16 ELISA and Ov16 RDT according to health districts

In the Doume Health District, all those who were positive (5/461) with Ov16 ELISA were also positive with Ov16 RDT and all negatives (456/461) with Ov16 giving a percent positive agreement (PPA) of 100.0 % and percent negative agreement (PNA) of 100.0 %. The Lomie Health District the Ov16 ELISA and Ov16 RDT agreed on 69 positives out of the 72 detected by Ov16 ELISA and 70 detected by the Ov16 RDT. All those who were negative for Ov16 RDT were also negative by Ov16 ELISA. There disagree just on one negative result, giving a PPA and PNA of 95.8 % and 99.9 % respectively. For the Nguelemendouka Health District, 422 participant’s samples were compared and both test agreed perfectly on 3 positives and 419 negatives, giving a PPA of 100.0 % and PNA of 100.0 %. In the Nwa Health District, 834 participants were examined, they agreed on 205 positive and 597 negative giving a PPA and PNA of 89.5 % and 98.7 %. All participants who were positive and negative with ELISA were also positive and negative with RDT in the Yagoua Health District. The PPA and PNA was 100.0 % and 100.0 % respectively. In the Yokadouma Health District, 908 participants were tested none was positive for both test and all 908 agreed on the negatives. The PPA was 0.00 % and PNA was 100.0 % (figure 4)

#### 3.5.4 Overall agreement (ORA) in the positives and negatives between Ov16 RDT and Ov16 ELISA by Health District

**Figure 5:**
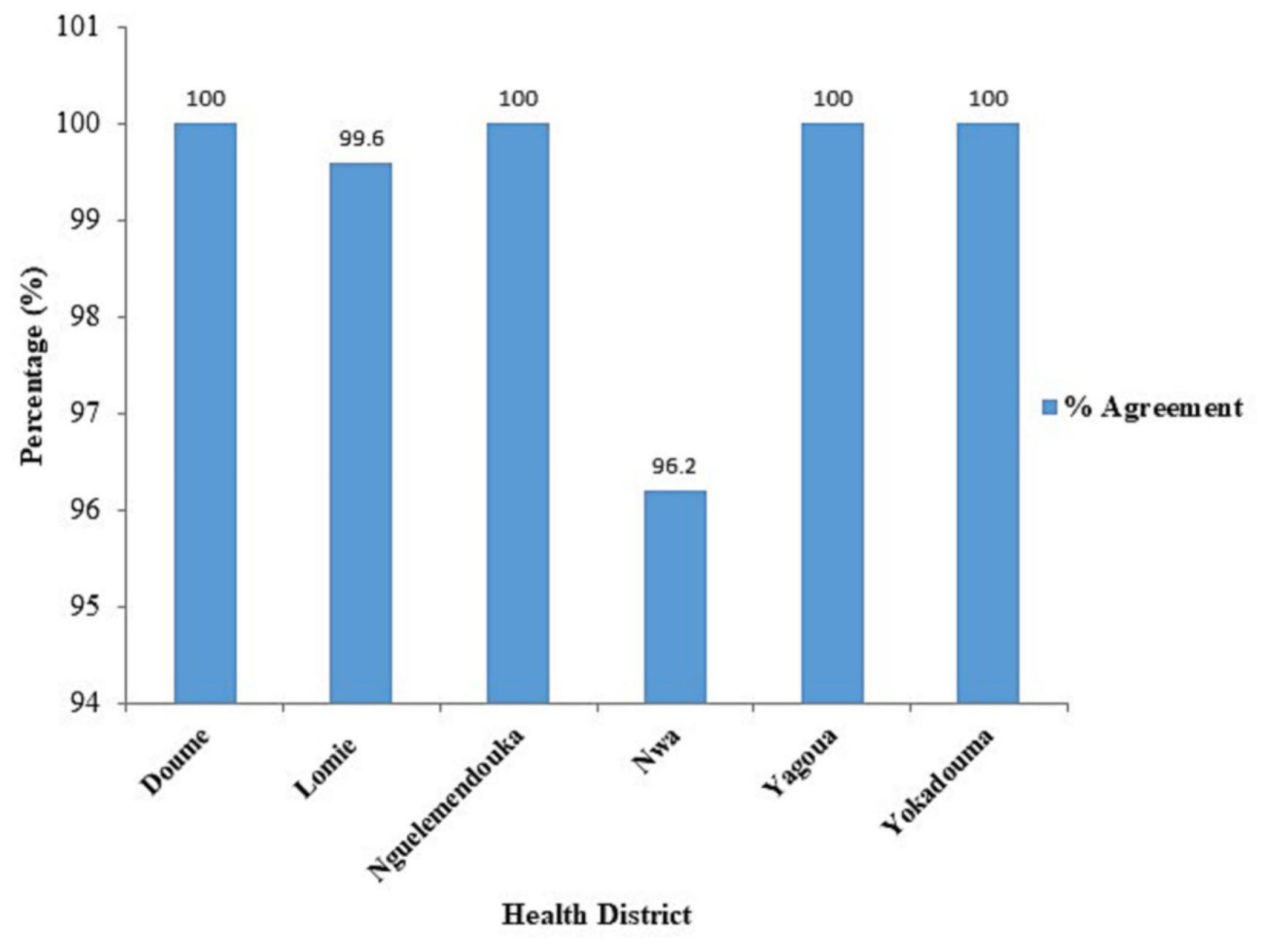
Overall Percentage agreement in test results between Ov16 RDT and Ov16 ELISA by health district

Figure 5 Shows that, of the 461 participants screened from the Doume Health District for onchocerciasis using the Ov16 rapid diagnostic test and the dried blood spots for ELISA, 5 agreed on positive result and 456 on negative results giving an overall percent agreement of 100.0 % for both tests. In a similar manner in the Lomie Health District, the agreement in positive and negative was 69 and 831 respectively for both tests giving a total percent agreement score of 99.6 %. Tests agreement were on 3 positives and 831 negatives in the Nguelemendouka Health District and the percent agreement was 100.0 %. The Nwa Health District agreed in 205 positive and 597 negative with a total percent agreement of 96.2 %. The Yagoua Health District agreed on 1 positive and 886 negative and the overall percent agreement was 100.0 %. In the Yokadouma Health District none agreed for positive test results and 908 for negative test results. The total percent agreement was 100.0 %.

#### 3.5.5 Influence of previous ivermectin treatment on the level of test agreement

In the Doume Health District, all the participants who said they had never taken ivermectin agreed positive for both tests (**P<0.001**). In a similar manner, 58 participants agreed positive by the both test from the Lomie HD among those who had taken ivermectin and only 14 with RDT and 12 with ELISA detected positive who had never taken ivermectin (**P<0.001**). In the Nguelemendouka HD, all participants who were positive for both test are those who had never taken ivermectin (**P<0.001**). The situation was different in the Nwa HD where, 203/229 positive were those who had taken ivermectin and only 26/229 positive had never taken ivermectin with the rapid test and ELISA also had a similar trend 190/213 positive had taken ivermectin and only 23/213 had never taken ivermectin (**P<0.001**). In the Yagoua HD, all the positive for both test where those who said they had never taken ivermectin (**P<0.001**). The Yokadouma HD, no positive and negative in relation to ivermectin treatment was detected (**P<0.001**; table 6).

## 4 Discussion

Findings from this study reveal that onchocerciasis is prevalent in the study communities with a sero-prevalence of 6.6 % using the rapid diagnostic test and 7.0 % with ELISA. The sero-prevalence was higher in the Health Districts of Nwa 25.5 % and 27.5 % with Ov16 RDT and Ov16 ELISA respectively. This high prevalence suggests that onchocerciasis is still a problem in the rural communities despite treatment with ivermectin MDA. The high prevalence detected in this communities could be due to some challenges faced during the implementation of control strategies such as difficulties in access, cultural barriers and preference for traditional methods of treatment A recent study conducted in the Bioko Island by Hernández-González *et al*., 2016[17] reported a similar sero-prevalence (7.9 %). Therefore there is a major concern that onchocerciasis may re-emerge as a major health problem for inhabitants of this districts. It is therefore necessary to set up well-planned strategies for the control using improved diagnostic methods to facilitate surveillance activities, monitoring and evaluating control efforts.

The sero-prevalence was higher in males than in females using both diagnostic methods 6.9 % with Ov16 RDT and 7.5 % with Ov16 ELISA. The difference in the infection rate according to sex may be due to endemicity, occupational exposure, and to some extent on the susceptibility of individuals. Although both males and females engaged in farming, women are better dressed and therefore, there is less exposure of large pa-rts of their bodies especially the lower limbs to black fly bites. Also, most men stay more outside the house than women, and hence have a greater exposure to bites by vector black flies. These findings contradicts studies carried out by Surakat *et al*., 2015[18] where the seroprevalence was higher in females than males because females were more engaged in agricultural activities than men in Nigeria.

Among the different age groups, prevalence was highest in the 41-50years age group, and duration of 40-60 years in the community. This indicates that onchocerciasis infection rate increases gradually with advancing age and increase duration in the community. Wogu and Okaka 2008[19] showed that individuals in the 60-69 years age group not only accounted for the second highest prevalence of symptoms of the disease but also exhibited all the symptoms. Those ≥ 70 years had the highest rate of infection (68 %), while participants 5-9 years (age group) had no symptoms of infection. Children born after the implementation of treatment are supposed to be antibody free because they are not exposed to the infective larvae, thus the reason children were excluded from our study population. These infections in these age groups (40-50), often result in major socioeconomic liabilities, as working-aged adults are often the ones debilitated, leaving the young to care for them as well as to provide for the family[20].

The overall percentage agreement of the Ov16-based tests was 99.2 % and the Kappa score was 0.936 which was statistically significant **P<0.001**. The kappa score was 0.936. Kappa quantifies the extent to which the observed agreement that the observers achieved exceeds that which would be expected by chance alone, and expresses it as the proportion of the maximum improvement that could occur beyond the agreement expected by chance alone. The kappa score indicates an excellent agreement between the two tests. In addition, the sensitivity of 91.3 % and specificity of 99.8 % also indicates that the rapid diagnostic test Ov16 is highly sensitive and specific in identifying IgG4 antibodies against *O. volvulus* antigens, The Ov16 antigen as a specific an early marker for the diagnosis of *O. volvulus* infections and can detect individuals with current and past infections[7,12, 21]. The Ov16 RDT test is a sensitive, specific, and very convenient means of detecting antibody provides rapid result and does not require expensive equipment for collection of serum samples or transportation of specimens to central laboratories for testing. Unlike microscopy or PCR, antibody tests do not provide definitive proof of current infection with sexually mature *O. volvulus* parasites. Antibodies to Ov16 may indicate the presence of current mature infections, pre-patent infections, past infections, or even heavy exposure to *O. volvulus* without establishment of mature infections. Despite this limitation, our results suggest that the Ov16 RDT test could be useful in the clinical setting for confirming the diagnosis in persons suspected of having onchocerciasis. As part of the global elimination program, Ov16 rapid diagnostic test would therefore be a useful rapid screening tool for determining the prevalence and distribution of *O. volvulus*. As reported previously by *Golden et al*., 2013 [7], the Ov16 rapid diagnostic test and Ov16 ELISA could detect all individuals with antibodies against *O. volvulus* infections. We found that the Ov16 RDT could detect fewer individuals with *O. volvulus* infection than the Ov16 ELISA (Table 3). In addition, all individuals positive with the Ov16 RDT were also positive for the Ov16 ELISA. The Ov16 ELISA could identify more positive than Ov16 RDT because, the Ov16 ELISA monoclonal antibody (mAb) could recognize more epitopes compared to the Ov16 RDT. Furthermore, the test protocol of the Ov16 ELISA includes series of incubations steps to release antibodies possibly trapped inside the immune complexes, which increases the chances of ELISA detecting more positives.

While the global elimination program of onchocerciasis is ongoing, highly sensitive and specific diagnostic assays are necessary to monitor and control the program. More importantly, the test has great potential as a tool for national and regional efforts to eliminate onchocerciasis. Firstly, antibody testing should be useful as a primary surveillance tool for efficiently assessing infection prevalence rates in untreated communities. Second, the RDT test may be useful for monitoring the success of control programs that aim to interrupt transmission. Serial surveys of sero-prevalence in young children or studies of sero-conversion rates in children may be practical and useful approaches to this pressing problem. Finally, detection of antibodies in children by the card test may be valuable as a tool for detecting residual foci of transmission and for certifying elimination of onchocerciasis in countries or regions that are in the later stages of elimination programs. This study demonstrated the value of the Ov16 RDT as a point-to-contact diagnostic tool and as a means of monitoring and evaluation of onchocerciasis transmission and post MDA surveillance especially when children are used as the sentinel population for TAS surveys [5,7]The Ov16 serologic test has been used to monitor progress toward elimination of river blindness in America and more recently in Africa[8]. Recent studies showed that filarial antigen tests are better alternative methods in diagnosing *Onchocerca volvulus* infections as they detect both past and current infections and could be used to monitor and verification of transmission in endemic areas following mass drug administration. The Ov16 ELISA has a higher performance in detecting *O. volvulus* infections than the Ov16 RDT test. In conclusion, our experience with the SD BIOLINE Oncho/Lf IgG4 biplex rapid test (based on the detection of IgG4 antibodies to recombinant antigen Ov16) was favourable. However, the Ov16 RDT test may be more appropriate to use in remote areas because of its ease of use and can be easily transported, it requires less blood (10 μL finger-prick blood) and results are obtained after 30 minutes making it more convenient for field application.

## 5 Conclusions

The results obtained indicate that both tests detected individuals with IgG4 antibodies against Ov16 antigen. Moreover, the Ov-16 sero-prevalence increased with age and increase duration in the community. The Ov16 RDT and Ov16 ELISA had an excellent agreement with an overall kappa score of 0.936 and an overall percent agreement of 99.2 %. The outcome of both test were associated with history of treatment with ivermectin.

## Abbreviations

APOC: African Programme for Onchocerciasis Control
CDTI: Community Directed Treatment with Ivermectin
CI: Confidence Interval
CNEC: Cameroon National Ethics Committee
DALY: Disability Adjusted Life Years
DEC: Diethylcarbamazine
ELISA: EnzymeLinked Immunosorbent Assay
FBS: Fetal Bovine Serum
GPS: Global Positioning System
HD: Health District
HRP: Horseradish Peroxidase
ID: Identification
IgG: Immunoglobulin gamma
IgG4: Immunoglobulin gamma subclass 4
L3: Larva stage three (infective larvae) Lat Latitude: Long: Longitude
MDA: Mass Drug Administration
Mf: Microfilaria(e)
NPV: Negative Predictive Value
O. v: Onchocerca volvulus
OD: Optical Density
ORA: Overall Agreement
Ov16: Onchocerca volvulus antigen 16
PATH: Program for Appropriate Technology in Health
PBS: Phosphate Buffer Solution
PBST: Phosphate Buffer Solution-Tween
PNA: Percent Negative Agreement
PNA: Percent Negative Agreement
POC: Point-of-care
PPA: Percent Positive Agreement
PPA: Percent Positive Agreement
PPV: Positive Predictive Value
RDT: Rapid Diagnostic Test
REFOTDE: Research Foundation for Tropical Diseases and Environment
TMB: Tetramethylbenzidine
WHO: World Health Organisation

## Figures and Tables

**Table 1:** Distribution of Sex, Age and duration in the community of the study participants

Variables	Frequency (N°)	Percentages (%)
**Sex**		
Males	2036	46.1
Females	2380	53.9
**Total**	**4416**	**100.0**
**Age group (years)**	**38.70±18.326(Mean ±SD)**	**Range(17-101)years**
17-30	1896	42.9
31-40	734	16.6
41-50	654	14.8
51-60	518	11.7
61+	614	13.7
**Total**	**4416**	**100.0**
**Duration in the community (Yrs)**	**30.77±20.236(Mean ±SD)**	**Range(5-101)years**
5-15	994	22.5
16-25	1218	27.6
26-35	648	14.7
36-45	519	11.8
46-55	415	9.4
56-65	289	6.5
66+	333	7.5
**Total**	**4416**	**100.0**

**Table 2:** Sero-prevalence of onchocerciasis using the Ov16-based diagnostic test according to health districts

Study Area (Hd)	Ov16 RDT		Chi square **P-value**	Ov16 ELISA		Chi square **P-value**
	POS/ TOT	% (95% CI)		POS/ TOT	% (95% CI)	
Doume	5/461	1.1 (0.15-2.05)		5/461	1.1 (0.15-2.05)	
Lomie	70/904	7.7 (5.43-8.77)		72/904	7.9 (6.14-9.66)	
N’ka	3/422	0.7 (-0.1-1.5)	**<0.001**	3/422	0.7 (0.16-1.24)	**<0.001**
Nwa	213/834	25.5(22.54-28.46)		229/834	27.5 (24.47-30.53)	
Yagoua	1/887	0.1 (-0.11-0.31)		1/887	0.1 (-0.11-0.31)	
Yakadouma	0/908	0.0(0.00)		0/908	0.0 (0.00)	
**Total**	**292/4416**	**6.6 (3.75-9.45)**		**310/4416**	**7.0(6.25-7.75)**	

**POS:** Positive test results, **TOT**: Total number of participants, **N’ka**: Nguelemendouka

**Table 3:** Prevalence of onchocerciasis using the Ov16 rapid test and Ov16 ELISA according to socio-demographic characteristics of study participants

Sex	**Ov16 RDT**		Chi square **p-value**	**Ov16 ELISA**		Chi square **p-value**
	POS/TOT	% (95% CI)		POS/TOT	% (95% CI)	
Males	140/2036	6.9(5.8-8)		152/2036	7.5(6.36-8.64)	
Females	152/2380	6.4(5.42-7.38)	0.514	158/2380	6.6(5.6-7.6)	<0.284
Total	292/4416	6.6 (5.87-7.33)		310/4416	7.0(6.25-7.75)	
***Age group (Yrs)***						
17-30	97/1896	5.1(4.11-6.09)		103/1896	5.4(4.38-6.42)	
31-40	67/734	9.1(7.02-11.18)		69/734	9.4(7.29-11.51)	
41-50	46/654	7.0(5.04-8.96)	**0.004**	51/654	7.8(5.74-9.86)	**0.004**
51-60	38/518	7.3(5.06-9.54)		42/518	8.1(5.24-9.36)	
61+	44/614	7.1(5.07-9.13)		45/614	7.3(5.24-9.36)	
Total	292/4416	6.6(5.87-7.33)		310/4416	7.0(6.25-7.75)	
***Duration in community (Yrs)***						
5-15	67/994	6.7(5.15-8.25)		70/994	7.0(5.41-8.59)	
16-25	72/1218	5.9(4.58-7.22)		83/1218	6.8(5.39-8.21)	
26-35	42/648	6.5(4.6-8.4)		44/648	6.8(4.86-8.74)	
36-45	42/519	8.1(5.75-10.45)	0.538	41/519	7.9(5.58-10.22)	0.872
46-55	22/415	5.3(3.14-7.46)		24/415	5.8(3.55-8.05)	
56-65	23/289	7.9(4.79-11.01)		24/289	8.3(5.12-11.48)	
66+	24/333	7.2(4.42-9.98)		24/333	7.2(4.42-9.98)	
	292/4416	6.6(5.87-7.33)		310/4416	7.0(6.25-7.75)	
***Previous intake of ivermectin***						
Yes	248/2340	10.6(9.35-11.85)		261/2340	11.2(9.92-12.48)	
No	44/2076	2.1(1.48-2.72)	**<0.001**	49/2076	2.4(1.74-3.06)	**< 0.001**
Totals	292/4416	6.6(5.87-7.33)		310/4416	7.0(6.25-7.75)	

**Table 4:** Cross tabulation of Ov16 RDT and Ov16 ELISA test results in the health district

Study area	Ov16 RDT and Ov16 ELISA test results			
	Ov16 RDT	Ov16 ELISA Results		
		Positive	Negative	Total
Doume	Positive	5	0	5
	Negative	0	456	456
	Total	5	456	461
% agreement = 100.0 %; Kappa score = 1.000, Chi square = P<0.001				
Lomie	Positive	69	1	70
	Negative	3	831	834
	Total	72	832	904
% agreement = 99.6 %; Kappa score = 0.969; Chi square = P<0.001				
Nguelemendouka	Positive	3	0	3
	Negative	0	419	419
	Total	3	419	422
% agreement = 100.0 %; Kappa score = 1.000; Chi square = P<0.006				
Nwa	Positive	205	8	213
	Negative	24	597	621
	Total	229	605	834
% agreement = 96.2 % ; Kappa score = 0.902; Chi square = P<0.001				
Yagoua	Positive	1	0	1
	Negative	0	886	886
	Total	1	886	887
% agreement = 100.0 % ; Kappa score = 1.000; Chi square = P<0.001				
Yokadouma	Positive	0	0	0
	Negative	0	908	908
	Total	0	908	908
% agreement = 100.0 %; Kappa score = 0.000				

**Table 5:** Cross tabulation of the overall agreement level between the Ov16-based diagnostic test

	Ov16 ELISA Test Results			
		Positive	Negative	Total
**Ov16 RDT Results**	**Positive**	283	9	292
	**Negative**	27	4097	4124
	**Total**	**310**	**4106**	**4416**
	**Overall % agreement 99.2 %, Kappa score 0.936, P< 0.001**			

**Table 6:** History of treatment with ivermectin and test agreement

Study area				
	Ov16 RDT	Ov16 ELISA Results		
		Positive	Negative	Total
Have taken	Positive	240	8	248
ivermectin	Negative	21	2071	2092
	Total	261	2079	2340
% agreement = 100.0 %; Kappa score = 0.936, Chi square = **P<0.001**				
Have not taken	Positive	43	1	44
ivermectin	Negative	6	2026	2032
	Total	49	2027	2076
% agreement = 99.6 %; Kappa score = 0.923; Chi square = **P<0.001**				
Total	Positive	283	9	292
	Negative	27	4097	4124
	Total	310	4106	4416
% agreement = 100.0 %; Kappa score = 0.936; Chi square = **P<0.001**				
